# Accumulation and contamination of gully pot sediments from varied land-use types: metal loads, concentrations and speciation

**DOI:** 10.1007/s11356-023-30062-1

**Published:** 2023-09-30

**Authors:** Snežana Gavrić, Kelsey Flanagan, Haoyu Wei, Heléne Österlund, Lian Lundy, Maria Viklander

**Affiliations:** Urban Water Engineering, Department of Civil, Environmental and Natural Resources Engineering, Luleå University of Technology, 971 87 Luleå, Sweden

**Keywords:** Urban stormwater runoff, Catch basin, Metal mobility, Sediment quality assessment, Sediment accumulation, Sediment loads

## Abstract

Urban stormwater typically enters sewer networks through gully pots, which allow a primary sedimentation of solids upstream of the piped network. The regular removal and disposal of retained sediment are necessary, costly and can involve environmental risks due to the contamination of sediments with substances from the urban environment such as metals. The concentrations and speciation of Cd, Cr, Cu, Ni, Pb and Zn were analysed in sediments from 26 gully pots located in different land use areas in Stockholm, Sweden. In addition, accumulation rates of both sediment and metal masses were evaluated, providing a basis for optimising maintenance practices and better understanding of impacts of characteristic urban land use types. Metal concentrations varied by at most a factor of eight between samples and were always below Swedish polluted site guidelines for less sensitive land use, with only eight samples exceeding the guideline values for Cu and Zn for sensitive land use. Sequential extraction showed Pb and Zn to be the most mobile metals. Sediment accumulation rates varied from 0.003 to 0.197 kg/m^2^ impermeable surface/year. Metal accumulation rates were much more variable than metal concentrations, with a factor of up to 172 between the highest and lowest rates and the highest metal accumulation rates corresponding to the lower range of mass loads in road runoff. Differences in metal concentrations, sediment or metal mass accumulations could not be solely attributed to either traffic or catchment land use. In contrast, traction grit used for winter road maintenance, which has low (but detectable) metal concentrations, is identified as a major component of gully pot sediments, with a combined effect of both moderating metal concentrations and contributing to total mass.

**Supplementary Information:**

The online version contains supplementary material available at 10.1007/s11356-023-30062-1.

## Introduction

Urban runoff generated on impervious surfaces is often directly drained into a piped network through gully pots (GPs) located along kerbs or in parking lots. GPs typically include a sump below the outlet providing the capacity to trap sediments and prevent their entrance into the piped system (Bertrand-Krajewski et al. 1993; Lager et al. 1977; Memon and Butler 2002a). As such, GPs can play an important role in stormwater management by preventing pipe blockages and reducing the loads of sediment (and associated pollutants) reaching receiving waters (Wei 2022).

As sediment accumulates in GP over time, it must be removed periodically to avoid GP-blockage-induced flooding and restore its retention capacity (Langeveld et al. 2016; Memon and Butler 2002b). Because sediment removal is costly (Post et al. 2017) and contributes to the environmental impact of urban drainage systems (Fathollahi and Coupe 2021), its frequency should be adapted to meet the needs of a given site. The rate at which the sump fills (sediment accumulation rate) depends on many factors, such as sump design, catchment characteristics, the mass of sediment available in the catchment, road maintenance activities and rainfall characteristics (Lager et al. 1977). Recent research has shown that GP sediment trapping efficiency also varies over the accumulation period in a given GP in relation to a range of factors, including the fullness of the sump, the position of inlet water jets relative to the GP outlet, the flow rate into the GP and the sizes of incoming particles (Rietveld et al. 2020a). However, in practice, GPs are often either emptied reactively when flooding occurs or at regular intervals (e.g. annually) determined a priori. Further knowledge on the rate of sediment accumulation in GPs under a variety of field conditions (e.g. in cold climate locations) is necessary in order to provide an evidence basis for optimising maintenance practices.

GP maintenance produces substantial quantities of sediment: e.g. dry mass of ca 870 ton/year for Zurich, Switzerland (Conradin 1989); a wet mass of ca 388 ton/year and 805 ton/year for North Shore City and Auckland City, New Zealand, respectively (Pennington and Kennedy 2008). This raise concerns both about the quality of accumulated sediments and practices for its disposal. Indeed, urban runoff is known to convey a range of contaminants from various sources (Müller et al. 2020), many of which are associated with the particulate phase (Zgheib et al. 2011) and thus accumulate with settled sediments. The levels of contamination of sediments accumulated in urban drainage facilities are likely to depend both on the particular sources present within a given catchment (Wei et al. 2022), as well as on the dillution of contamination by other sources of particles, such as soil, natural organic matter or traction grit (Gavrić et al. 2022). In this study, we focus on the quality of GP sediments with respect to metals due to an abundance of metal sources in the urban environment: traffic exhaust emissions, road, tyre and brake wear, as well as weathering from buildings (roof covers, downspouts) and street furniture (Petrucci et al. 2014).

Previous studies have compared metal concentrations in road dust (Birch and Scollen 2003) and GP sediments (Karlsson and Viklander 2008; Wei et al. 2022) from highly contrasting catchments, finding levels of contamination increased with increasing traffic density, i.e. from (< 2000 vehicles/day to > 20000 vehicles/day). In contrast, Pun et al. (2019) focused on contamination of sediments from GPs in areas with relatively high traffic (11,000–47,650 vehicles/day) and observed no clear increase in Cd, Cr, Cu, Pb, Ni and Zn concentrations in GP sediments with increasing traffic density but found an effect of land use (i.e. commercial > industrial > residential) for six studied metals.

Whilst the studies identified vehicular activities as a key source of sediments and associated contaminants in GPs, the influential factors on the loading and physicochemical properties of GP sediments are not limited to the traffic intensities alone but may also concern other characteristic catchment-activity features. Whilst earlier works have considerably contributed to a better understanding on the distribution of metals in, especially, GPs along trafficked roads and residential catchments, there is limited understanding of a combined evaluation of the loading, concentrations and speciation of metals with larger varieties of common land-use types.

The objective of this study is to characterise sediment accumulation rates, sediment quality (focusing on metal levels and speciation) and metal accumulation rates in 26 GPs located in Stockholm, Sweden, to provide the knowledge base necessary for establishing improved guidance on GP maintenance and sediment disposal. Various land use types in the urban catchment were selected (residential, parking lots, pedestrian walkways, commercial sites), and the sampled GPs were subjected to winter road maintenance work that includes the application of traction grit, a major source of sediments that is not present in the cities with temperate climate conditions. To the authors’ knowledge, it is the first study to combine an analysis of sediment quality with a calculation of sediment and metal mass accumulation rates in GPs, an approach that provides a better understanding of GP performance in trapping urban sediments and associated pollutants during long-term operation periods.

## Methods

### Study sites

Sediments were collected from 26 GPs in the City of Stockholm, Sweden where the average annual rainfall is 546 mm, calculated for the 30-year normal period 1991–2020 (SMHI 2022). The accumulation period of the studied GPs occurred between March 2020 and May 2021, when the precipitation in Stockholm was 710 mm (SMHI 2023a), out of which 44 days the air temperature was below zero Celsius (SMHI 2023b). Each GP’s catchment was delineated using the software Scalgo LIVE with topography data (LiDar data from a 2019 laser scan at a spatial resolution of 0.125 m × 0.125 m) provided by the City of Stockholm. The catchment delineation for each GP by different types of land covers (e.g. road and roof surfaces) suggested that the directly connected impervious surfaces to GPs comprised mainly trafficked road surfaces whilst only six GPs are characterised as directly connected to roof surfaces, accounting for 0.3–17% of the total directly connected impervious area (Table S8, supplementary material). The GPs were located in catchments with different land use types: commercial (com) [6], residential with apartment buildings (res) [8], residential with single detached houses (res_housing) [4], pedestrian and bicycle path (ped) [2] and parking lot (pl) [6].

Annual average daily traffic (AADT) and the percentage of heavy vehicles were available for six of the studied roads (City of Stockholm 2021). For the remaining 12 streets, traffic was estimated using the Swedish Transport Administration’s traffic generation tool (Swedish Transport Administration 2021) and measured values available for the surrounding streets. All study sites are in low-speed (< 50 km/h), low-traffic areas (< 4100 vehicles/day) where road maintenance activities include: (i) sweeping of streets twice/year, (ii) clearance of snow from the roads and parking lots as needed (approximately ten times per year) and (iii) application of a grit/salt mixture to improve traction on icy surfaces (250 g/m^2^ of grit/salt mixture per application, applied approximately ten times per winter). The grit material consists of one part aggregate size ≤ 8 mm to two parts aggregate size 2–6 mm mixed with salt (NaCl) to produce a salt concentration of 3% (City of Stockholm, pers. com).

GP IDs used throughout the paper consist of the land use type and traffic intensity, for example res1_3200 indicates a GP in a residential area and a road with an AADT of 3200 v/day. Full site-specific information can be found in the Supplementary Information.

### Sediment sampling

GP sediments were sampled during March and May 2021 representing accumulation periods of 314–389 days. To facilitate sediment sampling, supernatant water in GPs (where occurring) was pumped out at a maximum flow rate of 15 L/min. The pumping process ended when a sharp increase in the turbidity of discharged water was observed. The remaining water-and-sediment layer was considered to be a part of the sediment. The sediment was sampled using a stainless-steel scoop (FLEX-SYSTEM™) from one quarter column of the sediment bed to obtain the entire depth of the accumulated sediment. When the sediment bed was too compact to allow sampling, it was loosened using a vertical aluminium shovel (FLEX-SYSTEM™).

To obtain representative samples for physicochemical analyses, the collected sediments were first placed in two stainless steel trays (~ 3 L) for homogenisation. When possible, representative samples were collected following a coning and quartering approach and distributed to corresponding sample containers. However, the majority of the samples (23 of 26 samples) were not suitable for quartering due to a high water content; in this case, subsamples were collected using the spoon-based homogenisation approach previously described and adopted in Flanagan et al. (2021) (see Supplementary Information for a more detailed description of the method). Between each sampling location, the equipment was thoroughly rinsed with tap water and wiped with paper towels. Samples were placed in boxes with ice packs for same day transportation to the laboratory.

Besides the sediment samples, equipment blanks were collected by rinsing the equipment (trays, spoons and sampler) with approximately 1 L purified water after the equipment was cleaned following sampling. Assuming the leached metal mass in the blank sample (µg) to be spread through 3 L of sampled wet sediment, leached concentrations were negligible compared with the total concentrations measured in the sediment (at most 0.01% of the total concentrations).

### Analytical procedures

Sediment samples were analysed for both total concentrations and using five-step sequential extractions for six metals (Cd, Cr, Cu, Ni, Pb and Zn) by an accredited commercial laboratory (ALS Scandinavia AB, Luleå, Sweden).

For total analysis, sediment samples were oven-dried at 50 °C and dry-sieved (< 2 mm), followed by hot-block-assisted digestion in 7 M nitric acid prior to analysis. In parallel, a subsample was dried at 105 °C to enable correction of concentrations to the samples’ true dry weight. For the sequential extraction analysis, oven-dried and milled sediments were subjected to a five-step sequential extractions process following the method adapted from Hall et al. (1996a, b) (see Table 1). All samples were analysed for metal concentrations using ICP-SFMS adapted from ISO 17294–2:2016 and EPA-method 200.8:1994 and/or inductively coupled plasma atomic emission spectroscopy (ICP-AES) adapted from SS EN ISO 11885 and EPA-method 200.7.

**Table 1:** Fractions and reagents for the sequential extraction procedure

Step	Targeted fractionation	Reagents for extraction
I	Absorbed, exchangeable and carbonate-bonded metals	1.0 M acetate buffer (pH = 5)
II	Labile organic matter-bonded metals	0.1 M pyrophosphate solution (pH = 9)
III	Amorphous Fe/Mn oxides-bonded metals	0.25 M hydroxylamine hydrochloride
IV	Crystalline Fe-bonded metals	1 M hydroxylamine hydrochloride, 25% acetic acid
V	Stable organic forms and sulphides	Potassium chlorate, 12 M hydrochloric acid, 4 M nitric acid

Besides the GP sediment samples, a sample of the composite grit material was collected from the supplier. The grit material was crushed prior to total metal analysis following the same pre-treatment and analysis as GP sediments.

In addition to metal analyses, five basic parameters were measured, including pH, electrical conductivity (EC) [μS/cm], total organic carbon (TOC) [% DW], loss on ignition at 550 °C (LOI) [% DW] and particle size distribution (PSD). Determination of pH and EC involved immersing probes (WTW pH 300 and WTW 3110, respectively) in each sediment sample. TOC was analysed according to CSN ISO 10694 and CSN EN 15936 (acidification of sample to remove the total inorganic carbon (TIC) followed by a combustion in an oxygen-containing gas flow). LOI was analysed according to EN 15935:2012–11 after leaves were removed and the sample homogenised without prior sieving. PSD was determined using wet sieving for size fractions of > 63 mm, 31.5–63 mm, 16–31.5 mm, 8–16 mm, 4–8 mm, 2–4 mm, 1–2 mm, 0.5–1 mm, 0.25–0.50 mm, 0.125–0.25 mm and 0.063–0.125 mm. The size fraction < 63 µm was determined by the laser particle size analyser using liquid dispersion mode (CSN EN ISO 17892–4 and BS ISO 11277).

### Mass calculations and uncertainty propagation

The mass of dry sediment in each GP were calculated based on geometry measurements in the field and characterisation of the sediment in the laboratory (Eq. 1). The mass calculation for ped1_0 was, however, excluded as time period since the GP was last emptied was unknown. The masses of metals of interest (Cd, Cr, Cu Ni, Pb, Zn) accumulated in each gully pot were then evaluated according to Eq. 2. As in all field campaigns, the various terms used in these equations contain uncertainties due to the methods of sampling, sample mixing and separation, measurement and chemical analysis; these uncertainties have been propagated in order to quantify the uncertainties in the final masses determined using a Monte Carlo (MC) method.

The GP’s horizontal surface area (A_GP_) is calculated from the dimensions measured in the field (e.g. diameter). For GPs of standard dimensions (the case of 24 of the 26 GPs), the main source of uncertainty in this term was assumed to be tolerance in the fabrication of concrete structures. Two GPs were of nonstandard dimensions; in this case, uncertainty was assumed to be due to the measurements of the dimensions in the field. Prior to sampling and after pumping the standing water from the GP, the vertical aluminium shovel was pushed into the bottom of the GP and the depth of the sediment bed (${d}_{GP}$) was measured indirectly by measuring the water mark on the shovel; an uncertainty of + /– 0.03 m was assumed for each measurement to account for potential error in this indirect measurement. The average wet density (${\rho }_{s})$ of the sediments in each GP was calculated from the weight and volume of duplicate wet subsamples. The main source of uncertainty in this calculation was assumed to be due to the weighing and volume measurement. To calculate the mass of dry sediments, the wet sediment mass was multiplied by the sediment’s fraction of dry weight (DW); a main source of uncertainty in DW was assumed to be the mixing of the samples, which was evaluated by measuring the DW in six subsamples and considering the variability. A final source of uncertainty in this calculation was the representativity of the water content of the sampled sediment given the sampling method (i.e. due to collecting part of the wet sediment which often involved both a compact solid layer and a more liquid, sludge layer, the water content of the sample may differ from that initially in the gully pot); to account for this, it was assumed that the water content of the sampled sediment may differ by as much as + / − 20% from the whole quantity of sediment in the GP.

1 $${M}_{s,GP, dry} = {A}_{GP} *{d}_{GP}*{\rho }_{s}*DW$$

where ${\mathrm{M}}_{\mathrm{s},\mathrm{GP},\mathrm{ dry}}$ is the mass of dry sediment in each gully pot [kg], A_GP_ is the horizontal surface area of the gully pot [m^2^], d_GP_ is the depth of the sediment bed in the gully pot [m], ρ_s_ is the sediment density [kg/m^3^], DW is the fraction of dry weight in the sediment [unitless].

To enable comparison with other studies which focus on this fraction, metal analysis was carried out on sieved samples (< 2 mm) and the mass of metals calculated by multiplying the mass of dry, sieved sediment by the measured metal concentration. The fraction of particles smaller than 2 mm (${f}_{<2mm}$) was evaluated from the particle size distribution data and the main source of uncertainty was assumed to be the uncertainty associated with this measurement. The mass of dry, sieved sediment was then multiplied by the concentration of each metal, for which the main source of uncertainty was assumed to be the uncertainty associated with its analytical method.

2 $${M}_{metal} ={M}_{s,GP, dry}*{f}_{<2mm}*{C}_{metal}$$

where M_metal_ is the mass of a given metal in the gully pot [g], ${\mathrm{M}}_{\mathrm{s},\mathrm{GP},\mathrm{ dry}}$ is the mass of sediment in each gully pot [kg], f_<2 mm_ is the fraction of sediment sieved to 2 mm [unitless] and C_metal_ is the concentration of the metal [g/kg DW].

For the MC uncertainty propagation, each uncertain value used in the calculations was varied stochastically (i.e. randomly in a distribution assumed to underly the uncertainty). The list of varied parameters and distributions used is shown in Tables S1 and S2 in the Supplementary Information. The mass calculation was repeated 1,000,000 times; the median values and an empirically calculated 95% confidence interval representing lower and upper error bar (2.5 percentile and the 97.5 percentile) are used for analysis.

Estimated loads were further normalised by the accumulation period [year] and catchment impervious area [m^2^] to facilitate comparison between gully pots.

### Data analysis

Although most concentrations in this study could be quantified, total concentrations of Cd were below the limit of quantification (LOQ) in five samples. Regarding sequential extraction, analysis of all metals was < LOQ for some samples in Fraction 2 (labile organic form). It should be noted that when calculating of the shares of each fraction for sequential extraction, half of the LOQ is used, which is indicated in graphs with an asterisk next to the sample name. In the case of Cd, concentrations were below the LOQ in Fraction 2 in nearly all samples, making calculated proportions unreliable; as such, sequential extraction data on Cd has not been presented.

To identify correlations between, i.e. total metal concentrations, metal loading rates, total solid dry mass and normalised sediment accumulation rates, non-parametric statistical hypothesis tests were used where correlations were considered significant if the *p* value was ≤ 0.01. For uncensored parameters, the Spearman rho correlation tests were adopted; in the case of Cd, where total concentrations were left-censored, correlations were tested using the nonparametric Kendall’s tau test in the Non-detects and Data Analysis for Environmental Data package (NADA) in R. Significant differences between groups of samples (e.g. between normalised sediments accumulation rates, total metal concentrations and metal accumulation rates in GPs between different land uses) were also tested using non-parametric statistical hypothesis tests (Kruskal–Wallis test for non-censored data and the Peto-Peto generalised Wilcoxon test for censored data).

## Results and discussion

### Physicochemical characteristics of sediments

The pH of GP sediments (6.18–7.13) were similar to those reported in a previous study of GP sediments in Luleå, northern Sweden (Wei 2022), whilst EC values (1145 to 6430 µS/cm) were generally higher than those reported by Wei (2022), with the exception of GP samples res12_housing_30, ped2_0 and PL1, where EC values (130–351 µS/cm) were comparable. The higher EC values reported here likely reflect seasonal difference in sampling (i.e. spring in the present study, as opposed to autumn in (Wei 2022), providing an opportunity for salt to be washed out by summer rains). Moreover, studied GPs were located in Stockholm which has more frequent application of salt for winter road maintenance compared to Luleå, where sand and grit is the main strategy and road salt is only applied in for example, bus stops and main roads. TOC content ranged from 1.79 to 13.9% (DW) falling within the range reported previously for GP sediments, e.g. 0.4–39% (Jartun et al. 2008) and 9–23% (Fuchte et al. 2022). LOI values ranged from 1.6 to 29% (DW), whilst previous studies have reported LOI values for GP sediments of 2–5% DW (Karlsson 2009) and mean values of 17.9 and 15.4% reported for residential and city GP sediments, respectively (Grottker 1990).

Overall, GP sediment samples exhibited very variable size distributions with d_10_ of 0.006 – 0.35 mm (median of 0.04 mm), d_50_ of 0.04 – 5 mm (median of 0.9 mm) and d_90_ of 4 – 10 mm (median of 5 mm). Individual sediment samples also displayed highly heterogeneous size distributions with uniformity coefficients (defined as the ratio of d_60_/d_10_) of 10 – 267 (median of 30). This heterogeneity was also visible during the sampling process when sediment-size stratification along the in-pot vertical direction was observed in selected GPs such as ped2_0 and com4_2500 (coarser materials in the surface layer and finer particles in the bottom layer). Similarly, Irgang et al. (2001) reported a higher proportion of finer (< 0.075 mm) sediments in the lower part of the GPs.

**Fig. 1:**
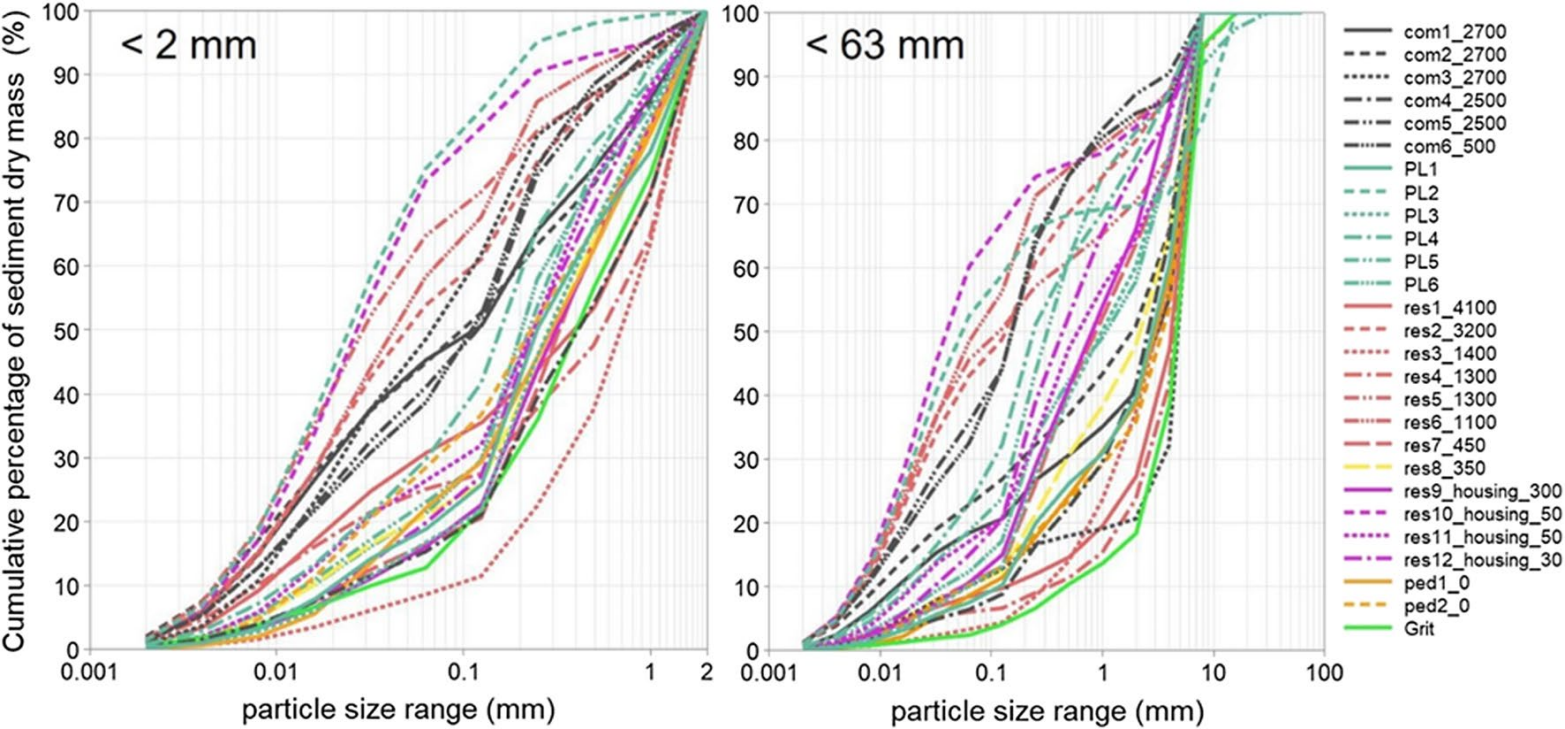
Particle size distributions presented as normalised to < 2 mm size fraction (left) and full-size range (< 63 mm) fraction (right) for gully pot sediments and traction grits used in the investigated catchments

A key challenge in interpreting sediment-dry-mass-based PSD analyses is the influence of larger grits, which may skew the PSD curves towards the coarser size fraction (Wei 2022). In this study, 4 – 8 mm sediments accounted for 5.4 – 68% (median of 23%) of total sediments in investigated GPs. To allow for identification of features associated with the fine-sized particles (< 2 mm, the fraction of interest for pollution management studies), the full-size-range PSD is normalised to < 2 mm size fraction. PSD for both the full-range (< 63 mm) and finer fraction (< 2 mm) of GP sediments are shown in Fig. 1.

Neither the PSD for < 63 mm nor < 2 mm sediments suggested a clear land-use-type influence; instead, the PSD curves are strongly GP-specific as even GP sediments from nearby catchments exhibited large PSD variations. This feature may be attributed to the generally low traffic intensities (< 5000) amongst the studied sites, as traffic is routinely considered to be an important factor determining physical sediment characteristics. GPs have an extremely localised drainage infrastructure, and thus the characteristics of sediments are a function of local processes and activities. An additional factor is the varied GP designs and catchment characteristics (provided in Supplementary Information), which may essentially contribute to the variations in in-pot hydraulic conditions.

As could be anticipated, comparison between the PSD of urban runoff suspended solids (SS) reported in the literature and GP sediment PSDs do not closely align. In contrast to the GP sediments of this study with a median d50 of 900 µm, the particles that are generally carried in road runoff appear to be much finer, with d50 of, i.e. 52.5 µm (Winston et al. 2023), 8–95 µm (Selbig et al. 2013), 11.7–102.9 (Charters et al. 2015), 29–300 (Kim and Sansalone 2008), 480–1200 µm (Sansalone et al. 1998) and 600 –1000 µm (Ellis and Harrop 1984) reported previously. This difference may reflect the reduced transportability of coarse particles as compared with fine particles (i.e. coarse particles entering GPs during dry weather or through bedload transport settle rapidly and remain in the GP, whilst fine particles more susceptible to resuspension and thus less efficiently retained. A further reason for observing coarser particles in many samples of the current study is the use of grit in Stockholm for winter road maintenance. However, further research is needed to fully understand the relationship between SS in runoff and GPs, e.g. to identify the mass and characteristics of sediments by-passing GPs.

### Metal concentrations of GP sediments

Total concentrations of selected metals in gully pot (GP) sediments from this study and from peer-reviewed studies are presented in Table 2. In the absence of specific guideline values for GP sediment disposal, concentrations are compared with the Swedish Environmental Protection Agency guidelines for contaminated soils developed to protect human health or the environment (SEPA 2009). All reported concentrations fell below the guideline values for soil quality of less sensitive land use, whilst eight samples exceeded values for sensitive land use for Cu (80 mg/kg DW) and/or Zn (250 mg/kg DW).

**Table 2:** Total metal concentrations [mg/kg DW] in < 2 mm gully pot sediments reported as median, range of concentrations (minimum–maximum), and mean (± standard deviation) in this study and previous research

Ref	AADT (number of GPs)	Land use type	Cd	Cr	Cu	Ni	Pb	Zn
Current study	< 4100 (*n* = 26)	Comm; Res; PL; ped	0.154 (< 0.1–0.418) [0.177 ± 0.0731]	25.0 (14.6–49.4) [28.9 ± 10.7]	40.5 (18.8–146) [54.8 ± 33.7]	15.8 (9.24–36.3) [16.9 ± 6.23]	14.2 (6.97–33.4) [16.6 ± 7.39]	154 (58.8–448) [188 ± 101]
Current study: grit (*n* = 1)			< 0.1	7.71	6.47	4.08	2.81	45.5
Wei (2022)	10,411 and < 500 (*n* = 7)	Road; Res	0.0874 (0.0742–0.334) [0.135 ± 0.0926]	25.8 (11.4–34.2) [26.7 ± 7.87]	41.1 (17.8–75.7) [43.2 ± 17.8]	12.2 (8.06–15.1) [12.5 ± 2.36]	11.0 (8.25–14.6) [11.4 ± 2.49]	135 (63.5–243) [146 ± 56.3]
Pun et al. (2019)	11,000–47,650 (*n* = 18)	Comm; Res; Ind	(0.2–7.9) [1.15 ± 1.82]	(14–439) [90.7 ± 99.3]	(27–1020) [186 ± 224]	(4–84) [25.2 ± 21.0]	(21–332) [110 ± 84.2]	(267–3700) [1615 ± 1138]
Karlsson and Viklander (2008)	25,500 and 500 (*n* = 10)	Road; Res	0.1 (0.1–0.3) [0.1 ± 0.1]	19 (8–31) [18 ± 7]	39 (10–81) [38 ± 21]	13 (4–20) [14 ± 6]	28 (12–67) [32 ± 19]	86 (25–121) [85 ± 31]
Jartun et al. (2008)	NA (*n* = 68)	Res; Ind; PL	0.42 (0.02–11.1) [1.13]	25 (11–135) [30]	97 (16–6600) [273]	24 (7.4–309) [32]	61 (9–675) [126]	403 (51.3–4670) [698]
Brown and Peake (2006)^a^	NA (*n* = 6)	Ind; Com; Res	NA	NA	[179 ± 145]	NA	[262 ± 167]	[424 ± 304]
Birch and Scollen (2003)	< 200–73,177 (*n* = 23)	Ind/Comm; Res	NA	(2–35) [20]	(47–398) [112]	(9–42) [20]	(62–827) [199]	(80–691) [257]
Grottker (1990)^b^	NA (*n* = 3)	Res; Road	17.62 (6.93–22.65) [15.7 ± 8.03]	56.5 (28.6–66.6) [50.6 ± 19.7]	497.7 (170.1–514.6) [394 ± 194]	94.3 (49.9–123.3) [89.2 ± 37.0]	1527 (444–1544) [1170 ± 630]	(895.7–2905.7) [2050 ± 1036]

Median (min–max)

[Mean + / − standard deviation]

^a^Six composite samples from 20 to 30 gully pots

^b^Total metal concentration in fraction < 1.6 mm

Overall, total metal concentrations in GP sediment observed in this study were at the lower end of values observed in previous studies. For example, whilst concentrations of Cr, Ni, Cu and Zn in the 26 GPs fall within the ranges reported previously, far higher maximum concentrations were reported by Grottker (1990), Jartun et al. (2008) and Pun et al. (2019). The mean Pb concentration was lower compared to those reported in previous research (see Table 2), with the exception of the study by Wei (2022) which reported comparable Pb concentrations. Total Cd sediment concentrations were similar to the values reported by Karlsson and Viklander (2008) and Wei (2022) but lower than other studies. One reason for the lower values observed in this study is likely to be the relatively low traffic intensity at the sites studied (< 4100 vehicles/day) as compared with the other studies which (where reported) often included gully pots from both low and higher traffic (e.g. AADT > 10,000 v/day) areas. A further reason for the relatively lower concentrations in the present study may be the dillution of traffic-related particles (with relatively high metal concentrations) by grit which contained lower concentrations. Indeed, for all metals, the metal concentrations determined in the grit were less than the lowest measured value in sediments, except for Cd which was not detected in all GP samples or in the grit. Whilst the Zn concentration in the grit was of the same order of magnitude as that in the least contaminated GP (45.5 vs. 58.8 mg/kg DW), grit concentrations of Cr, Cu, Ni and Pb were less than half the minimum concentration of the same metal in GP sediments, supporting the hypothesis that traction grits have a dilution effect.

In the present study, the highest levels of variation in metal concentrations were observed for Zn and Cu (where the maximum detected concentration was almost 8 times than the minimum value determined), followed by Pb, Ni and Cr (with ratios of maximum to minimum concentrations being 5, 4 and 3, respectively; Table 2). The ratio of Cd was > 4 (only a lower limit could be calculated due to the presence of non-detects). Whilst several previous studies similarly showed variations of less than an order of magnitude between GPs (Grottker 1990; Karlsson and Viklander 2008; Wei 2022), two previous studies showed variabilities between GPs in the range of 14–40, following the order Cd > Cr > Cu ~ Ni > Pb > Zn (Pun et al. 2019), and 12–555, following the order Cd > Cu > Zn > Pb > Ni > Cr (Jartun et al. 2008), indicating a need for further work to fully understand processes driving sediment metal accumulation rates.

**Fig. 2:**
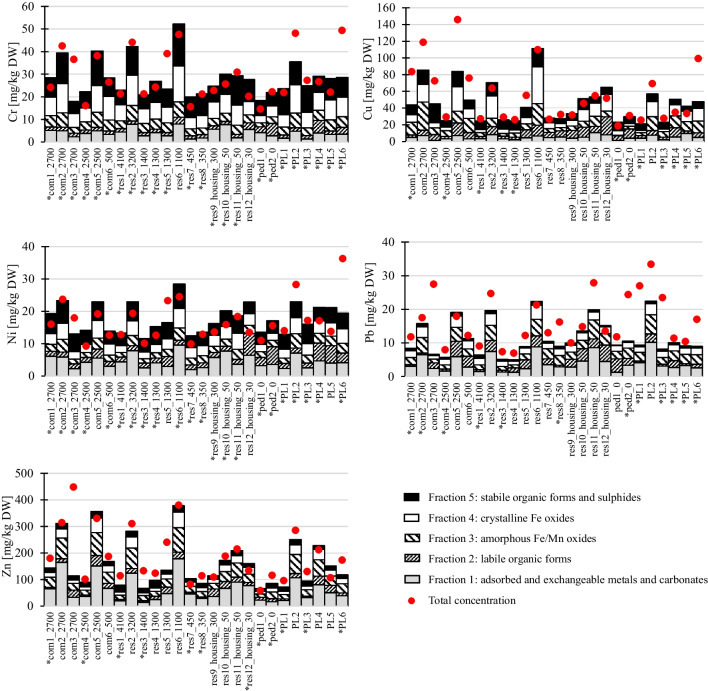
Speciation of metals among 5 fractions described as stacked bars. For samples with concentrations < LOQ in Fraction 2, Fraction 2 has been replaced by ½ LOQ; these samples are marked with (*). Total metal concentrations are described with red dots

All metals significantly and positively correlated with each other (Spearman rho = 0.52–0.91, *p* < 0.01 or Kendall’s tau 0.40–0.72, p < 0.01) except for Pb with Cu, Pb with Zn, Cd with Cr and Cd with Cu (see details in Figures S2 and S3 in Supplementary Information). The strongest correlations (rho or tau > 0.7) were observed within two distinct groups: Cr–Cu–Ni–Zn and Cd–Pb. One possible explanation for this could be that these groups of metals have similar sources in the studied catchments or that once released have similar patterns of environmental behaviour. All of these metals are associated with traffic (Müller et al. 2020), and because the main part of the impervious areas directly connected to GPs were trafficked surfaces, the correlation between the metals in GPs (located in commercial, residential and pedestrian catchments) and the traffic intensity was investigated. The analysis shows no correlations between metal concentrations in GPs and traffic intensity, nor was there a significant difference in metal concentrations by land use type (see Fig. 2 where total metal concentrations are identified using a red dot per GP). These results contrast previous studies of GP sediments, which found significantly higher metal contamination at sites with higher traffic intensity (Karlsson and Viklander 2008; Wei et al. 2022) and significantly different levels of contamination for different catchment types (commercial > industrial > residential) (Pun et al. 2019). This is likely explained by the relatively small variations in traffic between sites with differing land uses in the present study (i.e. difference of 3750 vehicles/day between the highest and lowest AADT) as compared with the other studies (differences of 25,000, 9900, 36,600 vehicles/day in Karlsson and Viklander (2008), Wei et al. (2022) and Pun et al. (2019), respectively). Another explanation is that one or both of these groups has additional sources that affected metal concentrations.

Further analysis of the relationship between basic parameters (“Physicochemical characteristics of sediments” section) and total metals indicates that only Cd shows a weak significant positive correlation with organic content in sediments measured as LOI (Kendall’s tau = 0.37, *p* < 0.01). This is broadly in keeping with previous research by Jartun et al. (2008), which reported an absence of correlation between organic content (measured as TOC) and the metals Pb, Zn and Cd. This result indicates that although metals and organic matter occur together in particles produced on urban surfaces such as tire wear particles (Müller et al. 2020), their levels in GP sediments do not appear to be driven by the same sources. One reason for this may be the accumulation of large amounts of natural organic matter (e.g. leaves) in many GPs independently of any anthropogenic processes. In contrast, all metals (except Cd and Pb) demonstrated a positive significant correlation with the proportion of fine particles (< 0.063 mm) (rho = 0.65–0.72 and *p* < 0.01) which could be explained with increasing specific surface area (m^2^/g) of particles with the decreasing diameter (Gnecco et al. 2019). However, both of these observations contrast a recent study of metal contamination of urban stormwater pond sediments in Sweden, which found LOI to correlate with total concentrations of Cd, Cu and Zn as well as with a global indicator of environmental risk due to metal contamination but found no correlations between total metal concentrations and the fraction of fine particles (Gavrić et al. 2022). It should be noted however that the methods used for determining the fraction of fine particles (< 0.063 mm) in the present study (i.e. wet sieving) differ from the laser diffraction particle size analyser used in Gavrić et al. (2022). Nevertheless, this indicates that the types of particles settling in GPs, as well as the drivers of sediment metal contamination, are different in GPs (upstream facilities with relatively short retention times) than in stormwater ponds (downstream devices with longer retention times).

### Mobility of metals in gully pot sediments

Figure 2 shows total metal concentrations (dry weight) in the < 2 mm fraction (indicated by the red dots) together with the results of the sequential extraction (as stacked bars; shading/hatching refers to extraction fractions) of un-sieved, milled sediments. Results from sieved and un-sieved samples are hence not directly comparable. However, it is noted that samples where total metal concentrations exceed those derived from summing the concentrations associated with each sequential extraction indicates that larger size fractions (i.e. > 2 mm) are effectively diluting metal concentrations (see earlier discussion in “Physicochemical characteristics of sediments” section).

Analysis of metal fractionation by sequential extraction (see graphs of relative distributions in Supplementary Information) showed that in the 26 GP samples, on average 36% of Zn and 32% of Pb were extracted in Fraction 1, which includes adsorbed and exchangeable metals and carbonates. Fraction 1 is the most mobile fraction, composed of metals that are easily leached under altered ambient environment, e.g. change of pH (Stone and Marsalek 1996). Moreover, Fraction 5, the least readily available fraction associated with stabile organic forms and sulphides, accounted for the lowest mean share of the total extracted concentrations, 14% and 6% for Zn and Pb respectively. In road runoff, Zn and Pb are recognised to have different speciation: Zn is typically more dissolved whilst Pb has a higher affinity for the particulate fraction (Sansalone and Buchberger 1997; Huber et al. 2016). However, sequential extraction data from the literature suggest that in the particulate phase, Zn and Pb have similar speciation to that in this study, with a higher share in Fraction 1 and lower share in Fraction 5, compared to the rest of the studied metals. Wei (2022), applying the same five step-extraction procedure to sieved GP sediment samples (< 2 mm), similarly reported Zn and Pb, to have high abundance amongst studied metals in Fraction 1 (mean 29–43%). Acid mobilisation of metals may occur between the rain events in gully pots (Morrison et al. 1988), in which case metals associated with Fraction 1 may transfer from GP sediments to the GP water. It should be noted, however, that the Pb concentrations in this study were not high (Table 2), and the maximum concentrations of Pb extracted in Fraction 1 was only 10 mg/kg DW. As for the remaining metals, Cr showed the lowest levels of mobility with the highest proportion in Fraction 5 (mean for 26 GPs: 34%), followed by Ni (28%) and Cu (26%).

### Mass loads

#### Mass loading of sediments

**Fig. 3:**
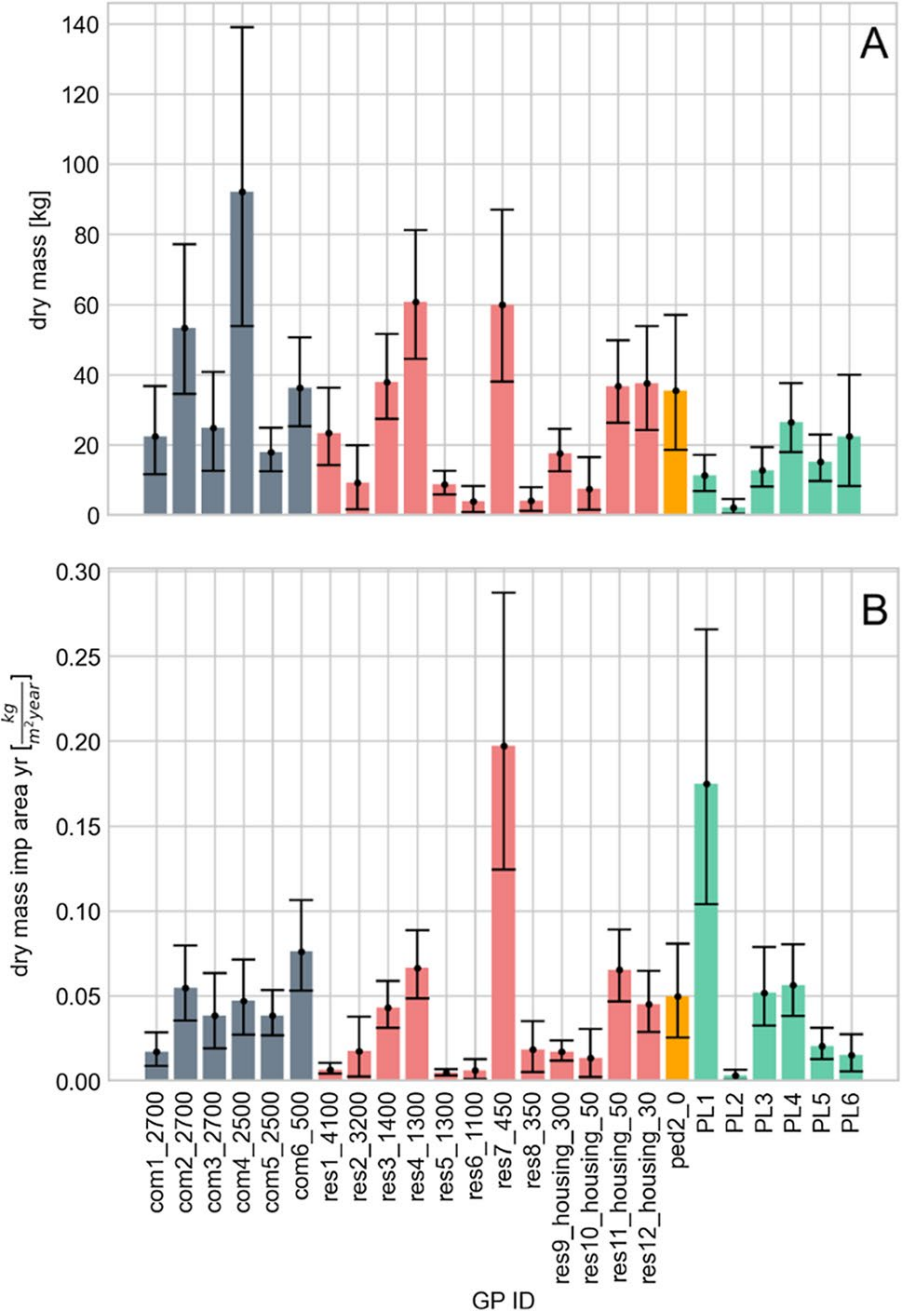
Total sediment dry mass (**A**) and normalised sediment accumulation rates by gully pots (**B**). Black dots and error bars indicate median values and their 95% confidence intervals developed based on a Monte Carlo simulation. All displayed confidence intervals originate from the uncertainty in the measurements and their propagation, described in the method section

Overall, total sediment dry mass varied from 2 to 91 kg as presented in Fig. 3A. Relative uncertainties (given as a 95% confidence interval) in each mass estimation, calculated from the MC approach, ranged from [− 27%, 34%] to [− 86%, 125%] depending on the GP (presented as error bars in Fig. 3).

No positive significant correlation was identified between the total solid dry mass and GP cross-sectional area nor impervious drainage area. These findings are not consistent with the positive relationship observed between the impervious drainage area and, for example, sediment bed volume (Rietveld et al. 2020b) and also sediment-bed-height-based accumulation rate (Post et al. 2016). Two factors contributing to these differences are identified. Firstly, the positive correlation between solid accumulation rate and catchment area was essentially attributed to the higher runoff volume associated with a larger contributing catchment area in the Rietveld et al. (2020b) study. However, such a relationship is only valid if the investigated GPs are situated within the same or adjacent catchments with similar rainfall event characteristics. Further the GPs investigated in Rietveld et al. (2020b) and Post et al. (2016) are more closely located (i.e. in the same or adjacent streets or nearby catchments). In contrast, the 26 GPs included in this work are widely distributed in various locations throughout Stockholm, and it is unlikely the specific local rainfall conditions were identical for all GP sites throughout the accumulation period. The second reason concerns the varied catchment types included in this work. As discussed in Rietveld et al. (2020b), the mass of solids retained in a GP is not only dependent on the size of the drainage area but is also a function of the mass availability and characteristics of solids on catchment surfaces, both of which are strongly related to catchment activities and processes. Thus, the integrated impacts of catchment-based activities and catchment characteristics may generally hinder the identification of correlation between solids mass and individual factors between and within mixed-land use sites.

To facilitate a cross-site comparison, total GP sediment dry mass was normalised by impervious catchment area and accumulation period. The normalised sediment accumulation rates are presented in Fig. 3B and a large variation between the investigated GPs is exhibited. Although some error bars clearly do not overlap indicating significant difference in median sediment accumulation rates between individual GPs both within and between catchment types, no significant differences (*p* = 0.579) were observed when grouping by catchment type nor was a significant correlation (*p* = 0.49) identified between the normalised solids accumulation rate and traffic intensities.

The normalised solid accumulation rates in this study are of the same magnitude as those reported in the literature (Table 3). However, compared with the normalised solid accumulation rates reported for GPs in a trafficked road catchment investigated by Wei et al. (2022), values reported in this work are several magnitudes lower. This might be due to larger amounts of traction grits used in northern Sweden (where the Wei et al. (2022) study was situated) due to the harsher winter conditions. More specifically, a traction grit application rate of ca 2500 g/m^2^/winter is reported for catchments investigated in this study (City of Stockholm, pers. com), a figure over 50% lower than the 5445 g/m^2^/winter traction grit application rate reported in Luleå (Luleå municipality, per. com; site of the Wei et al. 2022 study). It is also noted that the solid accumulation rates reported by, e.g. Rietveld et al. (2021) and Ellis and Harrop (1984) should only be interpreted as the maximum-possible solid accumulation rate in GPs as the sediments were collected at the entrance of GPs without being subjected to the transport process through the GP system.

**Table 3:** Normalised solid accumulation rates reported in this work and literatures

Catchment type	Time span (year)	Solids loading rate (kg/(m^2^ year))	Sampling method	Reference
Commercial	0.9 – 1.1	0.017 – 0.076	Partially emptying GPs	This work
Residential		0.005 – 0.197		
Pedestrian		0.051		
Parking lot		0.003 – 0.175		
Highway	NA	0.001 – 0.04	^1^Nylon net at inlet	Ellis and Harrop (1984)
Trafficked road and residential	1	0.086 – 0.093	^3^Fully emptying GPs	Grottker (1990)
Residential	> 25	0.004 – 0.014	Fully emptying GPs	Karlsson and Viklander (2008)
Trafficked road	0.3 – 1	0.011 – 0.118		
Residential	2	0.002 – 0.110	^2^Nylon net at inlet	Rietveld et al. (2021)
Residential	2 – 13	0.009 – 0.043	Fully emptying GPs	Wei et al. (2022)
Trafficked road		0.176 – 0.819		

Abbreviation: *NA* not available

^1&2^Nylon nets with a minimum pore size of 63 µm and 50 µm respectively

^3^Both gully pots with slotted buckets and sumps

#### Metal loading

**Fig. 4:**
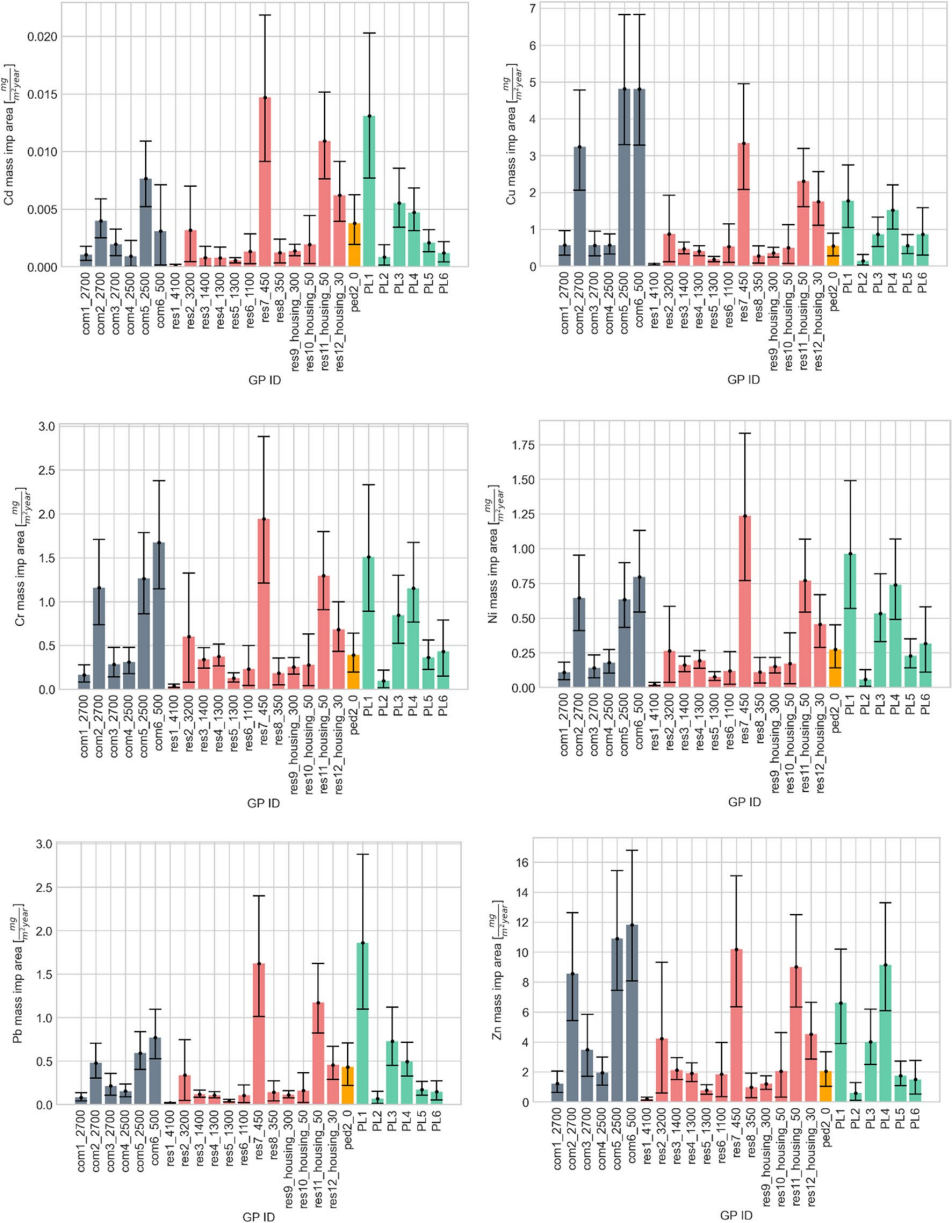
Mass of Cd, Cu, Cr, Ni, Pb and Zn normalised by catchment impervious area directly connected to the GP and accumulation period

Figure 4 shows that Zn had the highest metal loading rate (11.8 mg/m^2^/year) followed by Cu (4.82 mg/m^2^/year), Cr (1.94 mg/m^2^/year), Pb (1.86 mg/m^2^/year), Ni (1.23 mg/m^2^/year) and Cd (0.0147 mg/m^2^/year). Generally, higher levels of variation between GPs were observed for total metal loading rates compared to total concentrations; ratios of the maximum to minimum mass loadings were: 51 (Cr), 54 (Ni), 59 (Zn), 100 (Cu), 116 (Pb) and 172 (Cd). This higher variability is due to the very high variability in solid mass accumulation rates (a factor of 66 between the highest and lowest rate), as compared with variability in metal concentrations (factors of 3–8, depending on the metal). Further, whilst all metal loading rates (except for Cd) were significantly correlated with solid loading rates (*p* < 0.01), none correlated with metal concentrations, again due to the much higher variability in the mass accumulation rates. Moreover, contrary to the highest levels of variation observed in the case of total Zn concentrations between GPs, the Zn loading rate had the one of the lowest variations amongst studied metals. This may be explained by the fact that the sample with the lowest dry, sieved mass had a relatively high Zn concentration, whilst that with the highest mass had a relatively low Zn concentration (though there was no significant correlation between mass accumulation rates and total concentrations of any metals). For comparison, Cr had the lowest variation between GPs amongst metals in terms of both concentrations and loadings.

The relative uncertainties (95% confidence interval from the MC calculation) associated with metal loading rates were similar to those reported for total solid accumulation rates and ranged from [− 28%, + 37%] to [− 95%, + 152%]. As with solids loading rates, despite the high level of uncertainty, there were significant differences between metal loading rates in individual gully pots both within and between catchment types. For example, PL2 and res7_450 were the GPs with the lowest and the highest masses respectively in the case of Cr and Ni (Fig. 4). In the cases of Zn and Cu, similarly, sample PL2 had the minimum metal load whilst, although Res7_450 had a relatively high mass in case of Zn and Cu, the highest masses were estimated for two GPs located in the commercial catchments com6_500 and com5_2500. Pb showed a different trend; Fig. 4 suggests a more uniform distribution of the bars for commercial GPs, with samples res5_1300 and PL1 the two GPs that had the lowest and the highest accumulated metal mass, respectively (Fig. 4).

However, as was observed for the metal concentrations and solid loading rates, comparing three different land use types (commercial, residential and parking lots) showed no significant difference in metal masses normalised by impervious areas and accumulation period, nor there was a significant correlation with traffic intensity.

All metal loading rates (median values for each metal and GP from the MC calculation) correlated strongly with each other (Spearman rho 0.81–0.99, *p* < 0.01, see Figure S7 in Supplementary Information). These correlations are stronger than those between metal concentrations, due to less variable (and thus less site-specific) metal ratios in the GPs with relatively high metal mass loading rates, which are associated with a higher (< 2 mm) solid accumulation rates (see associated scatter plots in Supplementary Information). This suggests that the higher loading rates are associated with similar sources of particles at different sites. One hypothesis that may explain this observation is that traction grit is a major source of particles which dilutes site-specific metal contamination, so when large amounts of grit are present, metal ratios approach that of grit. Although at the time of its application, the majority of grit consists of coarse (> 2 mm) particles that would be excluded from total metal analysis and mass calculations, due to mechanical breakdown during use (e.g. crushed into smaller grits by vehicles), a larger proportion may be in this size range in the GP.

Previous research has demonstrated the impact of particle size fraction on the metal loading as a function of the specific area of the sediments (e.g. higher binding capacity for metals for smaller particles; Luoma and Davis 1983). The relatively larger specific area of the fine particles may favour metal uptake from the in-pot standing water as was observed in Morrison et al. (1988), leading to the further enrichment of metals on the finer particles. Similarly, Sansalone and Ying (2008) found that the hydraulically suspended fraction (< 25 µm) had higher metal concentrations than the settleable fraction (> 75 µm), though 90% of the total metal loading was associated with > 75 µm size fraction due to dominance in total mass and total surface area of sediments (Sansalone and Ying 2008). Although the limited hydraulic function of GPs challenges the retention efficiency for finer particles (Lager et al. 1977) resulting in medium-to-low metal concentrations in GP sediments, the large retention (up to 91 kg dry mass in one GP) of total sediments over a 1-year operation period may still significantly contribute to reducing the total metal loadings on the receiving waters. Additionally, leaching experiments on very coarse street sediments (250–2000 μm) by Borris et al. (2016) observed that coarse sediments can collect fine particles with attached metals available for later release under rainfall/runoff process. This highlights that as the coarse fraction, though less mobile, can leach metals over time, the management of these loads requires source control practices (e.g. effective street cleaning) and maintenance of drainage components (Sansalone and Ying 2008; Borris et al. 2016).

Annual runoff loads reported in the literature from different traffic areas (highways, parking lots and roads) were summarised by Huber and Helmreich (2016) as (median values): 35.5 mg/m^2^/year (range 3–378 mg/m^2^/year) in case of Cu, 11 mg/m^2^/year for Pb (range 5.5–50 mg/m^2^/year, including only studies from the twenty-first century) and 196 mg/m^2^/year for Zn (range 22–1900 mg/m^2^/year). Of these studies, the majority related to higher AADTs (up to 120 000 vehicles/day) compared with the present study. However, two studies of non-urban roads from 1979 to 1981 in WA, USA with comparable traffic density to catchments in the present study (2000 and 2500 vehicles/day) reported loads comparable to the higher loads identified in this study for Cu (3 and 6.5 mg/m^2^/year for Cu), though they were higher than those determined in this study for Zn (47 and 39 mg/m^2^/year) and are much higher for Pb (8 and 32 mg/m^2^/year, note that the relatively high values for Pb are likely due to the use of leaded fuel at the time of the study). This comparison indicates that some of the GPs in the present study trap metal loads in the same order of magnitude as loads produced on roads of similar traffic density, underlining the potential for GPs to help control pollutant loads from urban catchments. However, it should be noted that grit is likely an additional source of metal loading (albeit with a relatively low concentration) in the GPs in this study, which was likely not present in the non-urban catchments investigated in previous studies, which were located in a more temperate climate.

## Conclusions

This study investigated sediments in GPs from areas with low traffic (< 4100 vehicles/day AADT) and of a variety of land use types (residential, commercial, parking lots and pedestrian/bike walkways). Whilst metal concentrations varied by factors of 3–8 between the most and least contaminated, all metals investigated were below Swedish contaminated site guidelines for less sensitive land use and most were below the guidelines for sensitive land use, meaning that metal contamination does not limit the possibility of upland disposal of GP sediments.

The large variation in the accumulated dry mass of solids (2–91 kg) in this study calls into question the current practice of emptying all GPs at the same regular intervals. Moreover, the highest metal loading rates in GPs from this study were in the same order of magnitude for low-trafficked roads reported in previous studies. This suggests that in some cases, GPs may contribute to limiting the transport of metal loads from urban surfaces to receiving water, and as such have a potential role to play in the development of urban pollution mitigation strategies e.g. Programmes of Measures as required under the EU Water Framework Directive (2000). However, further research to evaluate the loads of both solids and metals transported into, accumulating within and exiting from GPs over time is necessary and should be done with uncertainty analyses to fully evaluate and compare the efficiency of GP at trapping metal loads and establish improved criteria for emptying GPs.

The variations observed in metal concentrations, solid accumulation rates or metal loading rates could not be directly attributed to either traffic or catchment land use factors. A major reason for this is likely the relatively limited variability in traffic rates in the present study. Another reason may be the effect of traction grit, which contributed to the solids accumulation in GPs along with other urban particles (i.e. tire wear particles). Indeed, both visual observations and particle size distributions point to the accumulation of grit in the GPs in this study providing new knowledge on the impacts of sediment sources diluting the metal concentrations whilst contributing to mass. Traction grit has relatively low metal concentrations (0.3–0.8 times the lowest metal concentrations observed in the GP) and has a high mass availability on the catchment areas (approximately 2.5 kg/m^2^/year on the impermeable surfaces in the study area, much larger than the highest solid accumulation rate in the GP). As such, it likely moderates metal concentrations, whilst contributing to both solid and metal mass accumulation processes in GPs. These effects may tend to mask differences in metal sources that may nevertheless exist between the catchments in this study.

### Supplementary Information

Below is the link to the electronic supplementary material.

Supplementary file1 (DOCX 355 KB)

## Data Availability

The data are available upon request.
