# Supplementary material for: Accumulation and contamination of gully pot sediments from varied land-use types: metal loads, concentrations and speciation
Source: Environ Sci Pollut Res Int. 2023 Sep 30;30(50):109825–40. doi: 10.1007/s11356-023-30062-1 (PMC10622363; doi:10.1007/s11356-023-30062-1)
Supplement: Supplementary file 1 — Supplementary file1 (DOCX 355 KB) [file 11356_2023_30062_MOESM1_ESM.docx]

Supplementary material

# 1. Spoon-based homogenisation approach:

Step one: Within each stainless tray where samples were collected, the sediment-water mixture was firstly well mixed with two spoons. The solid materials soon settled down and were divided into two rectangular zones (A, B, C and D in S 1). This step was done in both stainless-steel trays.


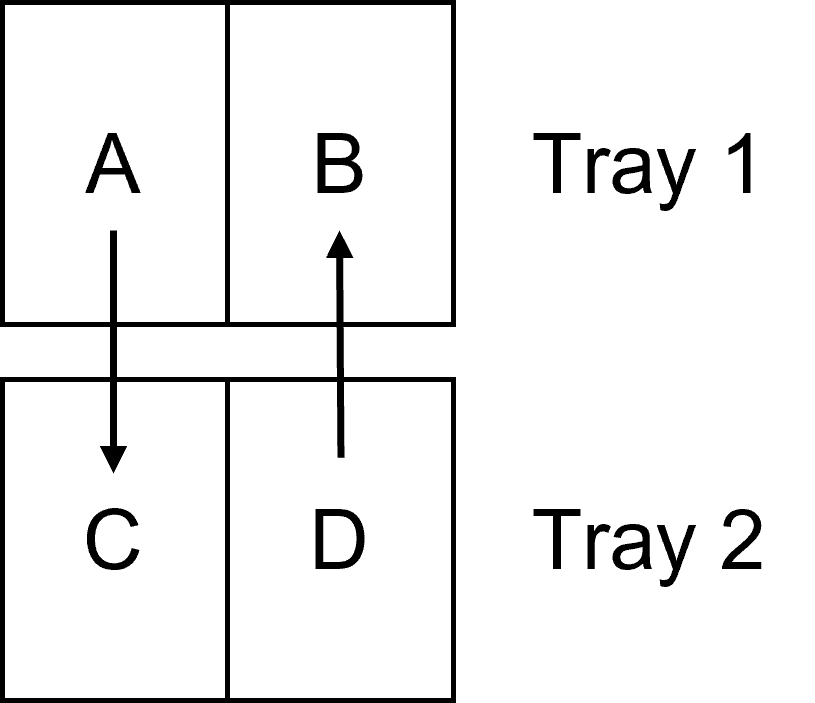


**Figure S1. Explanatory sketch of spoon-based homogenisation approach for deriving representative sediment samples.**

Step two: The second step commenced by exchanging the solid materials between two trays. An explanatory sketch is presented in S 1 where solids in A section of tray 1 is transferred to the C section on tray 2 and solids in B section of tray 2 is transferred to the B section on tray 1. After the transferring process, the sediment-water mixture in each tray is spoon-mixed again following the description in Step one. After a repetition of this step for twice, the sediment-water mixture in both trays is regarded as identical.

Step three: the sediment-water mixture is transferred to sample containers with the spoon in this step. All the sample containers are placed in a row with the lid removed. One spoon of the sediment-water mixture is consecutively transferred to each container for each round and a couple of rounds was taken until all sample jars were filled. When taking samples through the spoon, the spoon was firstly used to mix the sample so the sediments were in suspension.

# 2. Parameters and distributions used for the Monte Carlo uncertainty propagation for mass calculations

**Table S1: Summary of distributions used for the Monte Carlo calculation of the mass of dry sieved sediment in each gully pot.**

| **Term** | **Case** | **Stochastic component** | **Distribution type and parameters** |
| --- | --- | --- | --- |
| $A_{GP}$ | GP with round cross-section | Diameter | Uniform, min=49.4 cm, max=50.6 cm |
| $A_{GP}$ | GP with rectangular cross-section | Width | Uniform, min=width- 1 cm, max= width+ 1 cm |
|  |  | Length | Uniform, min=length-1 cm, max= length + 1 cm |
| $d_{GP}$ | Always | Sediment depth | Uniform, min=depth-3 cm, max=depth+3 cm |
| $\rho_{s}$ | Always | Mass of empty jar | Uniform, min=mass-1 g, max=mass+1 g |
|  |  | Mass of jar with sediments | Uniform, min=mass-1 g, max=mass+1 g |
|  |  | Volume of sediment in jar | Uniform, min=vol-12.5 mL, max=vol+12.5 mL |
| DW | Always | Dry weight | Normal, μ=mean of six measurements for each sample, σ=standard deviation of six measurements for each sample |
|  |  | Percent error in dry weight due to sampling | Uniform, min= -20%, max=20% |
| $f_{<2mm}$ | Always | Error in weight in each size fraction | Normal, μ=0, σ=5% |

**Table S2: Summary of distributions used for the concentration of each metal in the Monte Carlo calculation of each**

| Metal | Case | Stochastic component | Distribution Analytical Error |
| --- | --- | --- | --- |
| Cd | Concentration quantified | Error in concentration | Normal, μ=0, σ=5.6% |
|  | Concentration below limit of quantification | Concentration | Uniform, min=0, max=0.1 mg/L |
| Cr | Always |  | Normal, μ=0, σ=5.1% |
| Cu | Always |  | Normal, μ=0, σ=5.1% |
| Ni | Always |  | Normal, μ=0, σ=5.1% |
| Pb | Always |  | Normal, μ=0, σ=5.1% |
| Zn | Always |  | Normal, μ=0, σ=5.1% |

# 3. Data supporting analysis of correlations and significant differences with concentrations

| (a)   \|  \| Cr \| Cu \| Ni \| Pb \| Zn \| \| --- \| --- \| --- \| --- \| --- \| --- \| \| Cr \| 1 \| 0.72* \| 0.91* \| 0.52* \| 0.81* \| \| Cu \|  \| 1 \| 0.71* \| 0.37 \| 0.82* \| \| Ni \|  \|  \| 1 \| 0.66* \| 0.75* \| \| Pb \|  \|  \|  \| 1 \| 0.47 \| \| Zn \|  \|  \|  \|  \| 1 \| |
| --- | --- | --- | --- | --- | --- | --- | --- | --- | --- | --- | --- | --- | --- | --- | --- | --- | --- | --- | --- | --- | --- | --- | --- | --- | --- | --- | --- | --- | --- | --- | --- | --- | --- | --- | --- | --- |
| (b)  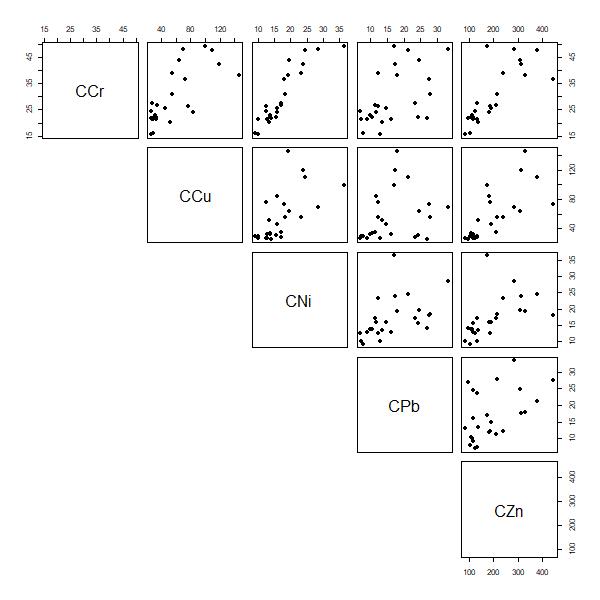 |

**Figure S2: (a) Correlation matrix (Spearman rho coefficients) and (b) scatterplots for concentrations of metals detected in all GP sediments with all concentrations in mg/kg DW. Significant correlations (P<0.01) are indicated with *.**

| (a)   \|  \| Cd \| \| --- \| --- \| \| Cr \| 0.37 \| \| Cu \| 0.33 \| \| Ni \| 0.52* \| \| Pb \| 0.72* \| \| Zn \| 0.40* \| |
| --- | --- | --- | --- | --- | --- | --- | --- | --- | --- | --- | --- | --- |
| (b)  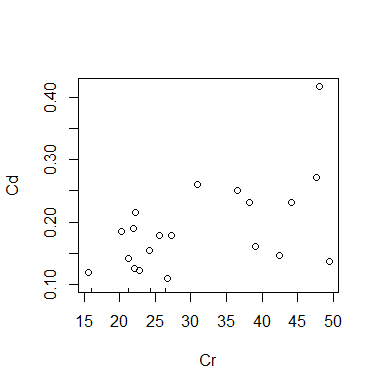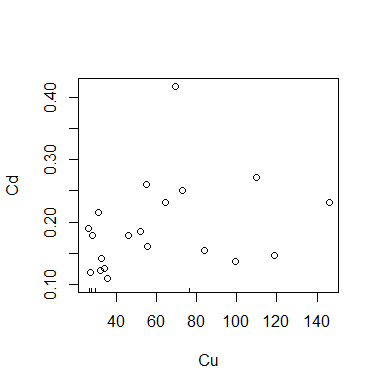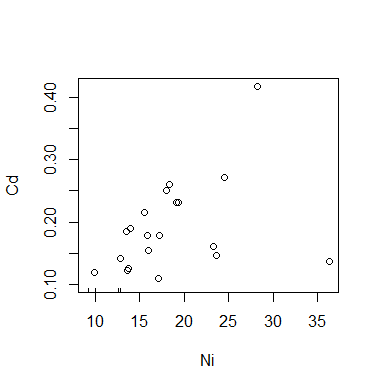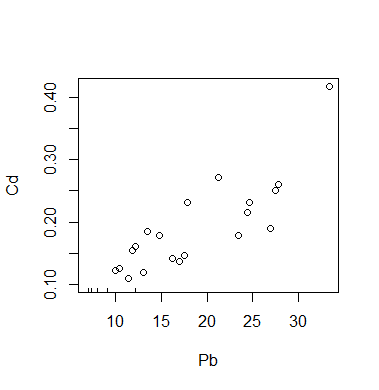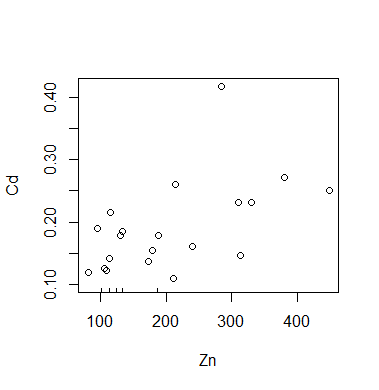 |

**Figure S3: (a) Kendall’s tau correlation coefficient for concentrations of Cd (for which not all concentrations were detected and data is censored) with other metals and (b) scatterplots for concentration of Cd vs other metals with all concentrations in mg/kg DW.**

**Table S3: Significant difference between metal concentrations for different land use (PL, comm, res)**

| Metal conc [mg/kg DW] | p | Kruskal-Wallis chi-squared |
| --- | --- | --- |
| Zn | 0.3146 | 2.3127 |
| Cu | 0.05558 | 5.78 |
| Pb | 0.4783 | 1.4752 |
| Ni | 2.3223 | 0.3131 |
| Cr | 0.4334 | 1.6724 |

|  | p | tau |
| --- | --- | --- |
| Cd and LOI | 0.00885 | 0.366154 |


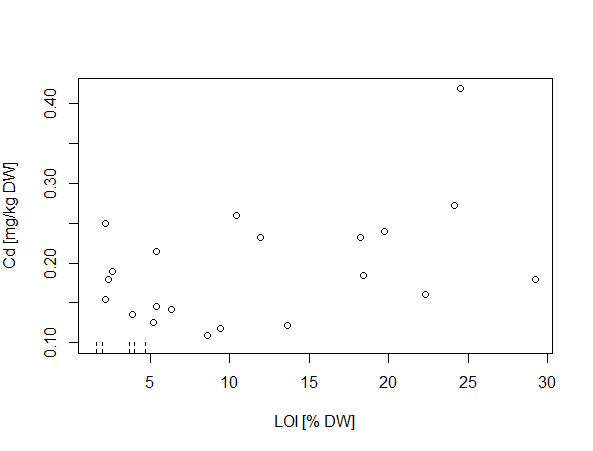


**Figure S4: Censored scatterplot and Kendall’s tau results for concentrations Cd&LOI that had significant correlation.**


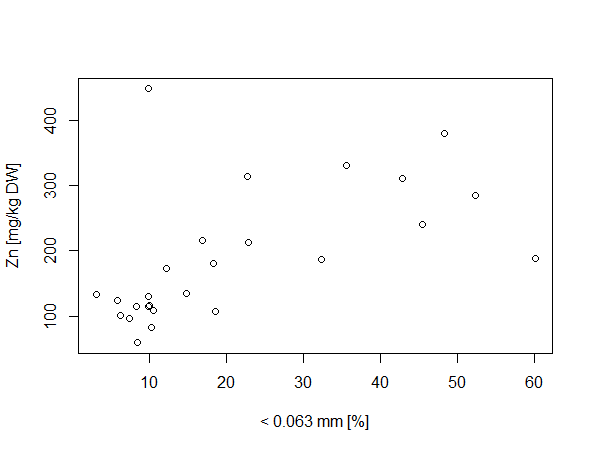

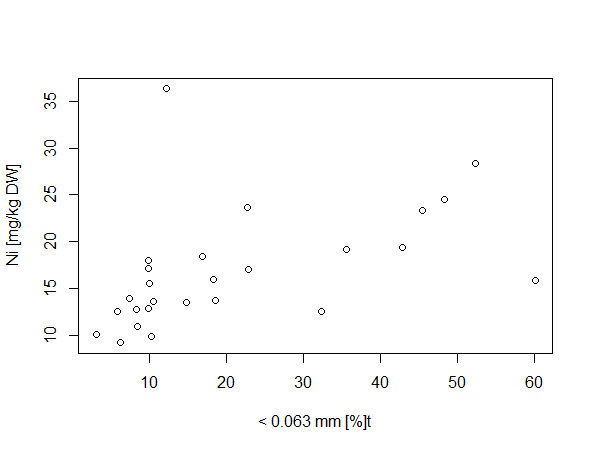


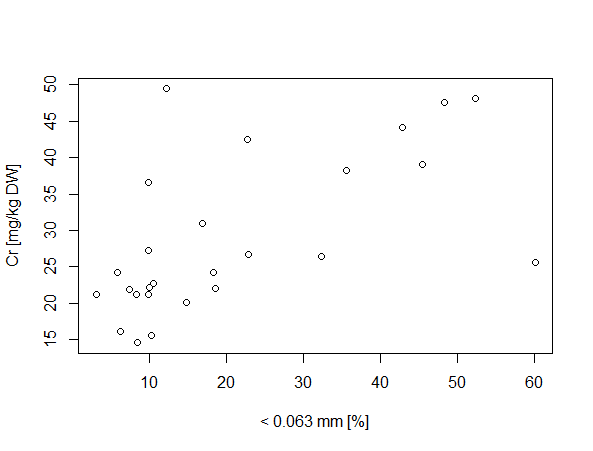


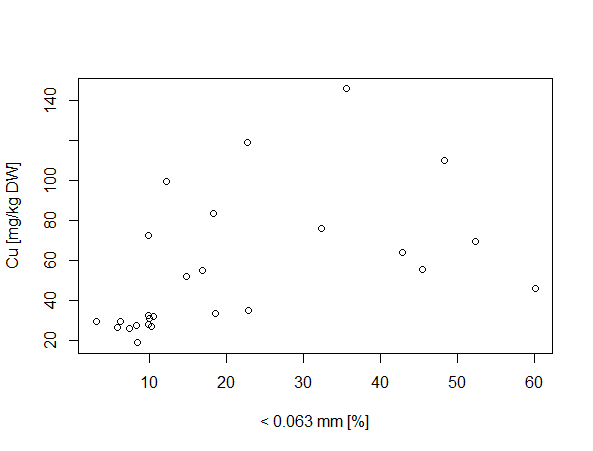


|  | rho | p-value (calculate it in R) |
| --- | --- | --- |
| Cr and Clay&Silt | 0.636 | 0.0005 |
| Cu and Clay&Silt | 0.715 | 0.0000 |
| Ni and Clay&Silt | 0.652 | 0.0003 |
| Zn and Clay&Silt | 0.649 | 0.0003 |

**Figure S5: Scatterplots and Spearman Rho test results for Cr, Cu, Ni and Zn**

#
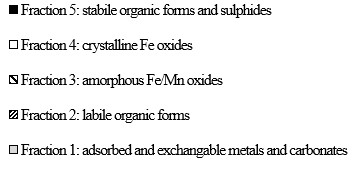
4. **
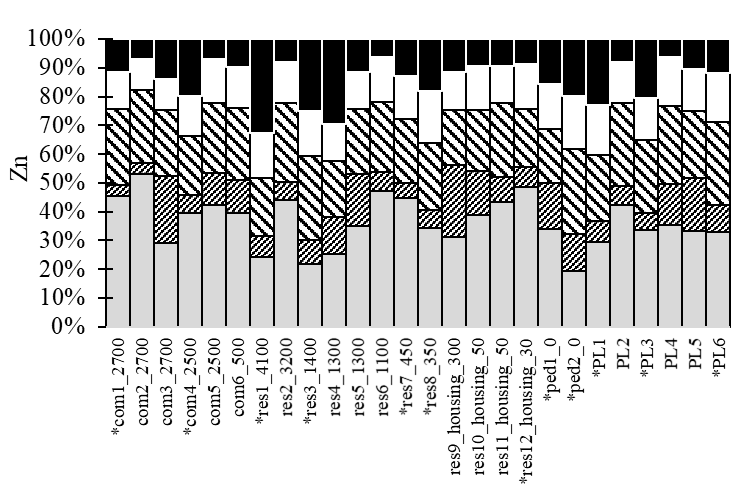

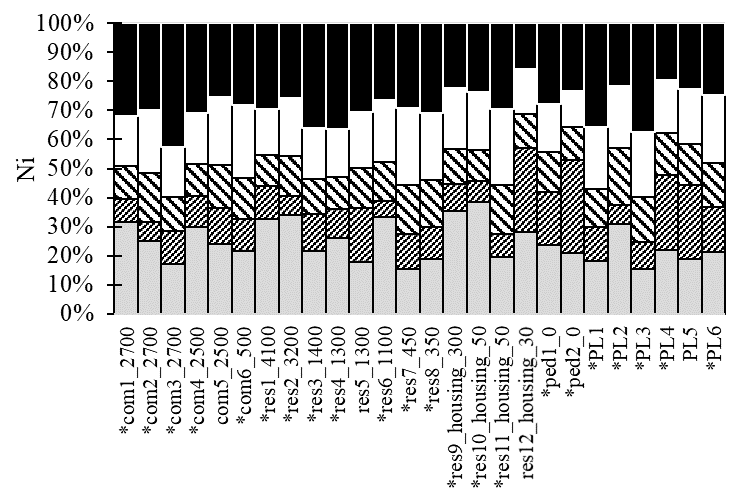

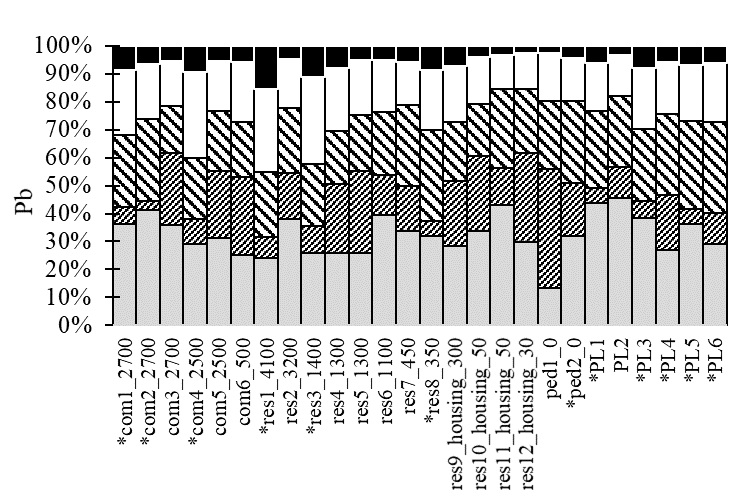
**
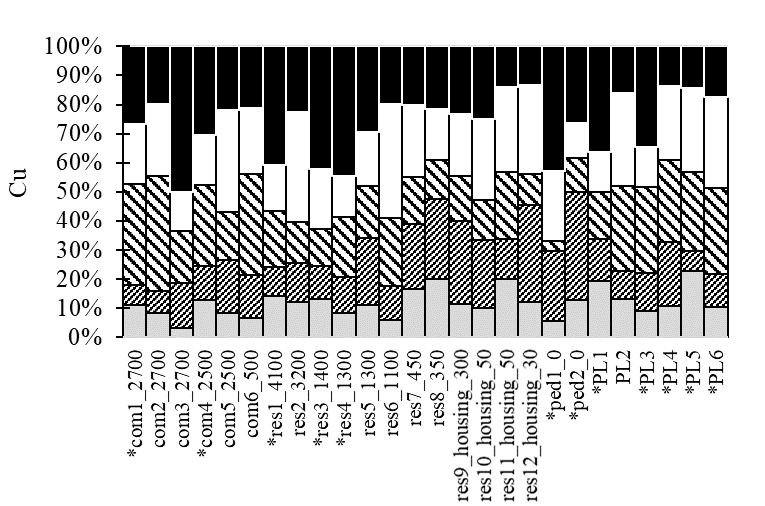
**
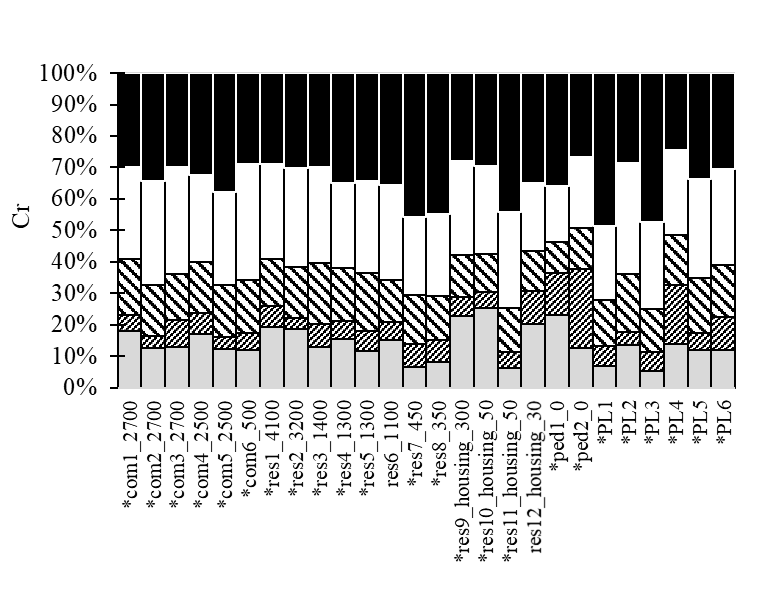
**Graphs of proportional speciation of metals in GP sediments

**Figure S6: Speciation graphs where in case metal is <LOQ, half of the LOQ is taken to make the graphs and calculate the sum of fractions.**

5. Data supporting analysis of correlations and significant differences with metal loading rates

| (a)   \|  \| Cd \| Cr \| Cu \| Ni \| Pb \| Zn \| \| --- \| --- \| --- \| --- \| --- \| --- \| --- \| \| Cd \| 1 \| 0.83* \| 0.81* \| 0.84* \| 0.93* \| 0.82* \| \| Cr \|  \| 1 \| 0.9* \| 0.99* \| 0.93* \| 0.92* \| \| Cu \|  \|  \| 1 \| 0.88* \| 0.86* \| 0.89* \| \| Ni \|  \|  \|  \| 1 \| 0.93* \| 0.88* \| \| Pb \|  \|  \|  \|  \| 1 \| 0.9 \| \| Zn \|  \|  \|  \|  \|  \| 1 \| |
| --- | --- | --- | --- | --- | --- | --- | --- | --- | --- | --- | --- | --- | --- | --- | --- | --- | --- | --- | --- | --- | --- | --- | --- | --- | --- | --- | --- | --- | --- | --- | --- | --- | --- | --- | --- | --- | --- | --- | --- | --- | --- | --- | --- | --- | --- | --- | --- | --- | --- |
| (b)  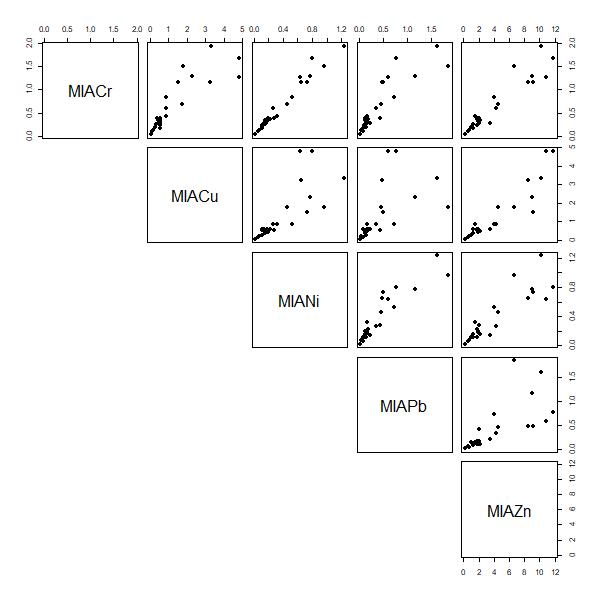 |

**Figure S7: (a) Correlation matrix (Spearman rho coefficients) and (b) scatterplots for normalized mass-loading rates in mg/(m^2^yr). Significant correlations (P<0.01) are indicated with *.**

**Table S4: Significant difference between metal masses (normalized per impervious area and accumulation period) for different land use (PL, comm, res)**

|  | p | Kruskal-Wallis chi-squared |
| --- | --- | --- |
| Zn | 0.3318 | 2.2067 |
| Cu | 0.1098 | 4.4183 |
| Pb | 0.3816 | 1.9267 |
| Ni | 1.605 | 0.4482 |
| Cr | 0.5134 | 1.3333 |
| Cd | 0.6188 | 0.96 |

**Table S5: Field observations**

| GP ID | Field observations |
| --- | --- |
|  |  |
| res1_4100 | sediments are gritty with few leaves and a bit greasy looking |
| res2_3200 | No standing water in GP lots of leaves |
| res3_1400 | Lots of leaves Sediment looked gravelly and not smelly |
| res4_1300 | / |
| res5_1300 | Lots of leaves |
| res6_1100 | / |
| res7_450 | Smelly sediments with many leaves and earthworms |
| res8_350 | Lots of leaves, earthworms and gravels |
| res9_housing_300 | Leaves on top layer |
| res10_housing_50 | Occurrence of one metal stick in the GP Mainly leaves in the GP |
| res11_housing_50 | / |
| res12_housing_30 | Lots of leaves Fine-sized sediment sludge |
| comm1_2700 | Lots of leaves in the sediments though no tree is around Lots of gravels on the road surface around |
| comm2_2700 | Oily standing water |
| comm3_2700 | Near a bus stop Lots of gravels and leaves |
| comm4_2500 | Oily sediments  Leaves on top, followed by gravelly sediments, followed by fine sediments |
| comm5_2500 | Near a construction site Loose black sediment with oily film |
| comm6_500 | Smells like sewage Oily standing water with floating of leaves |
| ped1_0 | Vegetation growing in the GP very deep sediment layer with unknown elapsed time since last emptying |
| ped2_0 | Gravels on the top layer with fine-sized sediments at the bottom Many earthworms |
| PL1 | Sediments are dry |
| PL2 | Many leaves and are smelly |
| PL3 | Really smelly sediments with lots of dead earthworms |
| PL4 | Liquidy, sandy and oily sediments Grease floating on the surface of standing water |
| PL5 | Lots of leaves |
| PL6 | Lots of leaves Greasy sediments |

**Table S6: Traffic and associated road maintenance activities**

| **GP ID** | **Traffic and associated road maintenance activities** | | | | | | | |
| --- | --- | --- | --- | --- | --- | --- | --- | --- |
|  | **Street layout (I - receives runoff from intersection or turning point;  PL - parking along the same side of the street where the gully pot is;  Carwash- exposed to possible street car washing )** | **Land use** | **AADT** | **Number of heavy duty vehicles** | **Number of lanes** | **Speed limit** | **Street Class** | **Street sweeping frequency** |
| res1_4100 | I;PL | residential apartments | 4100 (y. 2014) | 8% | 2 | 50 | Class 6 (Brun) | 2 times/year |
| res2_3200 | I;PL |  | 3200 (y. 2014) | 8% | 2 | 50 | Class 6 (Brun) | 2 times/year |
| res3_1400 | High slope street |  | 200 (y. 2014) | 8% | 2 | 50 | Class 6 (Brun) | 2 times/year |
| res4_1300 | / |  | 1300 | NA | 2 | 30 | Class 6 (Brun) | 2 times/year |
| res5_1300 | / |  | 1300 | NA | 2 | 30 | Class 6 (Brun) | 2 times/year |
| res6_1100 | / |  | 1100 | NA | 2 | 30 (50) | Class 2 (blå) | 1 time/week |
| res7_450 | I; combined drainage | residential detached house | 200 (y. 2014) | 8% | 2 | 30 | Class 6 (Brun) | 2 times/year |
| res8_350 | before road narrowing; combined drainage |  | 200 (y. 2014) | 8% | 2 | 30 | Class 6 (Brun) | 2 times/year |
| res9_housing_300 | I;PL;carwash |  | 200 (y. 2014) | 8% | 2 | 30 | Class 6 (Brun) | 2 times/year |
| res10_housing_50 | at the entrance to the parking from a house; carwash |  | 200 (y. 2014) | 8% | 2 | 30 | Class 6 (Brun) | 2 times/year |
| res11_housing_50 | combined drainage; carwash |  | 50 | NA | 2 | 30 | Class 6 (Brun) | 2 times/year |
| res12_housing_30 | I;carwash |  | 30 | NA | 2 | 30 | Class 6 (Brun) | 2 times/year |
| comm1_2700 | I; | commercial | 2700 (y.1998) | na | 2 | 50 | Class 6 (Brun) | 2 times/year |
| comm2_2700 | I; |  | 2700 (y.1998) | na | 2 | 50 | Class 6 (Brun) | 2 times/year |
| comm3_2700 | I;PL;before bus stop |  | 2702 (y. 2014) | 10% | 2 | 50 | Class 6 (Brun) | 2 times/year |
| comm4_2500 | I; PL; before the road narrowing |  | 2500 (y. 2014) | 20% | 2 | 50 (30) | Class 6 (Brun) | 2 times/year |
| comm5_2500 | I;PL |  | 2500 | NA | 2 | 50 | Class 6 (Brun) | 2 times/year |
| comm6_500 | I; stop and go for loading and unloading |  | 200 (y. 2014) | 10% | 2 | 50 | Class 6 (Brun) | 2 times/year |
| ped1_0 | away from the high traffic road | pedestrian area | / | / | / | / | District administrations responsibilety | NA |
| ped2_0 | below high traffic road |  | / | / | / | / | District administrations responsibilety | NA |
| PL1 | / | car park | 12848 visitors in October 2021 | NA | 2 | / | Class 6 (Brun) | 2 times/year |
| PL2 | / |  | 12848 visitors in October 2021 | NA | 2 | / | Stockholm Parking | Once a year (spring) and if necessary |
| PL3 | / |  | 12848 visitors in October 2021 | NA | 1 | / | Stockholm parking | Once a year (spring) and if necessary |
| PL4 | I;PL on the road |  | 5 832 visitors in October 2021 | NA | 2 | / | Stockholm parking | Once a year (spring) and if necessary. |
| PL5 | / |  | 5 832 visitors in October 2021 | NA | 2 | / | Stockholm parking | Once a year (spring) and if necessary |
| PL6 | at a curve |  | 5 832 visitors in October 2021 | NA | 1 | / | Stockholm parking | Once a year (spring) and if necessary |

**Table S7: Sediment accumulation time**

| **GP ID** | **Sediment accumulation** | | |
| --- | --- | --- | --- |
|  | **Date of last emptying** | **Date of sampling** | **Number of accumulation days** |
| res1_4100 | 01-03-2020 | 25-03-2021 | 389 |
| res2_3200 | 01-03-2020 | 25-03-2021 | 389 |
| res3_1400 | 01-03-2020 | 25-03-2021 | 389 |
| res4_1300 | 01-03-2020 | 23-03-2021 | 387 |
| res5_1300 | 01-03-2020 | 23-03-2021 | 387 |
| res6_1100 | 01-03-2020 | 25-03-2021 | 389 |
| res7_450 | 15-07-2020 | 25-05-2021 | 314 |
| res8_350 | 15-07-2020 | 25-05-2021 | 314 |
| res9_housing_300 | 01-03-2020 | 23-03-2021 | 387 |
| res10_housing_50 | 01-03-2020 | 23-03-2021 | 387 |
| res11_housing_50 | 15-07-2020 | 25-05-2021 | 314 |
| res12_housing_30 | 01-03-2020 | 23-03-2021 | 387 |
| comm1_2700 | 01-03-2020 | 25-03-2021 | 389 |
| comm2_2700 | 01-03-2020 | 25-03-2021 | 389 |
| comm3_2700 | 01-03-2020 | 24-03-2021 | 388 |
| comm4_2500 | 01-03-2020 | 25-03-2021 | 389 |
| comm5_2500 | 01-03-2020 | 24-03-2021 | 388 |
| comm6_500 | 01-03-2020 | 24-03-2021 | 388 |
| ped1_0 | 26-06-2020 | 27-05-2021 | 335 |
| ped2_0 | 26-06-2020 | 27-05-2021 | 335 |
| PL1 | 03-07-2020 | 25-05-2021 | NA |
| PL2 | 03-07-2020 | 25-05-2021 | 326 |
| PL3 | 03-07-2020 | 25-05-2021 | 326 |
| PL4 | 26-06-2020 | 27-05-2021 | 335 |
| PL5 | 26-06-2020 | 25-05-2021 | 333 |
| PL6 | 26-06-2020 | 27-05-2021 | 335 |

**Table S8: Catchment delineation**

| **GP ID** | **Catchment delineation** | | | | | | |
| --- | --- | --- | --- | --- | --- | --- | --- |
|  | **Catchment area total [m^2^]** | **Disconnected impervious surfaces [m^2^]** | **Perviousness land use type in Scalgo [m^2^]** | **Directly connected road [m^2^]** | **Directly connected roofs [m^2^]** | **Parking lot and other impervious surfaces (pedestrian/bicycle pathways) directly connected [m^2^]** | **Impervious catchment area directly connected [m^2^]** |
| res1_4100 | 8986.94 | 1476 | 4352.7 | 1425 | 0 | 1732.97 | 3160 |
| res2_3200 | 681.55 | 0 | 212.77 | 268.64 | 0 | 200.14 | 469 |
| res3_1400 | 902.33 | 0 | 111.1 | 402.98 | 0 | 388.25 | 791 |
| res4_1300 | 995.36 | 0 | 167.06 | 468.34 | 0 | 356.95 | 825 |
| res5_1300 | 1894.48 | 38.44 | 211.41 | 770.48 | 0 | 874.16 | 1640 |
| res6_1100 | 617.61 | 0 | 43.02 | 289.84 | 0 | 284.75 | 575 |
| res7_450 | 1567.91 | 0 | 1233.86 | 333.83 | 0 | 0 | 334 |
| res8_350 | 945.88 | 15.95 | 682.62 | 247.30 | 0 | 0 | 247 |
| res9_housing_300 | 6102.25 | 130.14 | 5043.52 | 440.28 | 3.05 | 485.27 | 929 |
| res10_housing_50 | 1691.52 | 64.02 | 1146.31 | 197.56 | 63.05 | 220.16 | 481 |
| res11_housing_50 | 3412.28 | 313.86 | 2481.36 | 255.50 | 102.03 | 259.98 | 618 |
| res12_housing_30 | 2381.77 | 102.92 | 1527.73 | 473.75 | 0 | 277.49 | 751 |
| comm1_2700 | 1166.05 | 0 | 8.24 | 716.77 | 0 | 441.05 | 1160 |
| comm2_2700 | 873.17 | 0 | 0 | 356.86 | 0 | 516.31 | 873 |
| comm3_2700 | 601.14 | 0 | 25.41 | 318.06 | 8.13 | 249.55 | 576 |
| comm4_2500 | 2168.25 | 205.25 | 204.00 | 360.00 | 0 | 1399 | 1760 |
| comm5_2500 | 3816 | 67.00 | 3329 | 283.00 | 28.00 | 109.00 | 420 |
| comm6_500 | 427.5 | 0 | 0 | 287.61 | 0 | 139.89 | 428 |
| ped1_0 | NA | NA | NA | NA | NA | NA | NA |
| ped2_0 | 11000 | 545.00 | 9706 | 0 | 0 | 780.00 | 780 |
| PL1 | 70.97 | 0 | 0 | 0 | 0 | 70.97 | 71 |
| PL2 | 758.75 | 0 | 0 | 0 | 0 | 756.75 | 766 |
| PL3 | 270.3 | 0 | 0 | 0 | 0 | 270 | 270 |
| PL4 | 514.36 | 0 | 0 | 401.69 | 0 | 112.67 | 514 |
| PL5 | 866.98 | 0 | 59.28 | 0.2 | 0 | 807.50 | 808 |
| PL6 | 1602.94 | 0 | 0 | 0 | 15.19 | 1588.16 | 1600 |

**Table S9: Gully pot designs**

| **GP ID** | **Gully pot designs** | | | | | | |
| --- | --- | --- | --- | --- | --- | --- | --- |
|  | **H of the sediment [cm]** | **Gully pot inner diameter [cm]** | **Road surface to sediment surface [cm]** | **GP depth (road surface to GP bottom) [cm]** | **top of the road to top of the outlet pipe [cm]** | **outlet pipe diameter [mm]** | **Sump depth [cm]** |
| res1_4100 | 12 | 50 | 167 | 179 | 128 | 200 | 31 |
| res2_3200 | 19 | 50 | 153 | 172 | 140 | 225 | 9.5 |
| res3_1400 | 17 | 50 | 126 | 143 | NA | 225 | NA |
| res4_1300 | 25 | 50 | 169 | 194 | 124 | 225 | 47.5 |
| res5_1300 | 12 | 50 | 175 | 187 | 130 | 225 | 34.5 |
| res6_1100 | 11 | 50 | 185 | 196 | 117 | 225 | 56.5 |
| res7_450 | 45 | Rectangle 60*40 | 152 | 197 | Outlet invisible | 225 | NA |
| res8_350 | 4 | 40 | 56-46 |  | 48 | NA | NA |
| res9_housing_300 | 16 | 50 | 173 | NA | 150 | 225 | NA |
| res10_housing_50 | 11 | 50 | 185 | NA | 129 |  | NA |
| res11_housing_50 | 32 | 50 | 165 | 197 | 140 | 225 | 34.5 |
| res12_housing_30 | 56 | 50 | NA | NA | NA | 225 | NA |
| comm1_2700 | 15, but still lots of water | 50 | 155, but still lots of water | 170 | 110 | 225 | 37.5 |
| comm2_2700 | 32, very liquidy muddy sediment | 50 | 170 | 202 | 145 | 225 | 34.5 |
| comm3_2700 | 17 | 50 | 128 | NA | 62 | NA | NA |
| comm4_2500 | 45 | 50 | 160 | 205 | 146 | 225 | 36.5 |
| comm5_2500 | 27 | Rectangle 60*37 | 170 | NA | Outlet invisible | NA | NA |
| comm6_500 | 23 | 50 | 180 | 203 | 148 | 225 | 32.5 |
| ped1_0 | > 125 cm but we only took top 20 cm layer | 50 | 65 | NA | Outlet invisible | 225 | NA |
| ped2_0 | 21 | 50 | 45-48 | NA | Outlet invisible | NA | NA |
| PL1 | 8 | 40 | 170-178 | 183 | NA | 160 | NA |
| PL2 | 11 | 40 | missing | NA | 270 | 150 | NA |
| PL3 | 10 | 40 | 230 | 240 | 210 | NA | NA |
| PL4 | 21 | 50 | 173 | 194 | 136 | 225 | 35.5 |
| PL5 | 11 | 50 | 175 | 186 | 140 | NA | NA |
| PL6 | 19 | 50 | 170 | 189 | 137 | NA | NA |
